# Comparison of worm development and host immune responses in natural hosts of *schistosoma japonicum*, yellow cattle and water buffalo

**DOI:** 10.1186/1746-6148-8-25

**Published:** 2012-03-13

**Authors:** Jianmei Yang, Zhiqiang Fu, Xingang Feng, Yaojun Shi, Chunxiu Yuan, Jinming Liu, Yang Hong, Hao Li, Ke Lu, Jiaojiao Lin

**Affiliations:** 1 Shanghai Veterinary Research Institute, Chinese Academy of Agricultural Sciences, Key Laboratory of Animal Parasitology, Ministry of Agriculture of China, Shanghai, People's Republic of China

## Abstract

**Background:**

Yellow cattle and water buffalo are two of the most important natural hosts for *Schistosoma japonicum *in China. Previous observation has revealed that yellow cattle are more suited to the development of *S. japonicum *than water buffalo. Understanding more about the molecular mechanisms involved in worm development, as well as the pathological and immunological differences between yellow cattle and water buffalo post infection with *S japonicum *will provide useful information for the vaccine design and its delivery procedure.

**Results:**

The worm length (*p *< 0.01), worm recovery rate (*p *< 0.01) and the percentage of paired worms (*p *< 0.01) were significantly greater in yellow cattle than those in water buffalo. There were many white egg granulomas in the livers of yellow cattle, but fewer were observed in water buffalo at 7 weeks post infection. The livers of infected yellow cattle contained significantly increased accumulation of inflammatory cells, and the schistosome eggs were surrounded with large amounts of eosinophil infiltration. In contrast, no hepatocyte swelling or lymphocyte infiltration, and fewer white blood cells, was observed in water buffalo. The percentage of CD4^+ ^T cells was higher in yellow cattle, while the percentage of CD8^+ ^T cells was higher in water buffalo from pre-infection to 7 w post infection. The CD4/CD8 ratios were decreased in both species after challenge with schistosomes. Comparing with water buffalo, the IFN-γ level was higher and decreased significantly, while the IL-4 level was lower and increased gradually in yellow cattle from pre-infection to 7 w post infection.

**Conclusions:**

In this study, we confirmed that yellow cattle were more suited to the development of *S. japonicum *than water buffalo, and more serious pathological damage was observed in infected yellow cattle. Immunological analysis suggested that CD4^+ ^T cells might be an integral component of the immune response and might associate with worm development in yellow cattle. A shift from Th1 to Th2 type polarized immunity was only shown clearly in schistosome-infected yellow cattle, but no shift in water buffalo. The results provide valuable information for increased understanding of host-schistosome interactions, and for control of schistosomiasis.

## Background

Schistosomiasis is one of the most serious parasitic zoonoses in the world. Approximately 365,770 people are currently infected with *S. japonicum *and the disease remains one of the most important public health problems in China [1]. About 46 species of mammal are known to carry natural infection with *S. japonicum*, such as rats, rabbits, dogs, cats, horses, cattle, sheep, donkeys, and monkeys, but only some of them are a source of human infection [2] Previous studies have revealed that the susceptibility of different types of host varies; for example, mice and yellow cattle are more sensitive than rats and water buffalo to infection with *S. japonicum *(Chinese strain)[3].

In China, the areas endemic for uncontrolled schistosomiasis are mostly in the marshland/lake and mountainous regions [4-6]. Epidemiological survey has revealed that domestic animals, especially bovines, play an important role in the transmission of schistosomiasis in these regions [7]: they are the most important reservoirs for schistosomes and are considered the major source of infection for human schistiosomiasis [8]. Two kinds of bovine reared commonly in these endemic regions are yellow cattle (*Bos taurus*) and water buffalo (*Bubalus bubalis*). These animals are naturally infected with schistosomes and spread more eggs into the environment than human and other animal hosts, and are considered to be the main sources of transmission of schistosomiasis in most lake and marshland endemic areas [9,10].

There is a significant difference in the rate of development between worms from yellow cattle and water buffalo infected with *S. japonicum*[11]. He et al. infected mice, rats, guinea pigs, rabbits, goats, sheep, pigs, water buffalo, yellow cattle, horses and another 12 kinds of animal with *S. japonicum *under identical conditions, and observed the development of parasites in these hosts for up to 60 weeks. The results showed that the development rates of *S. japonicum *in these hosts were quite different, with the highest infection rate of 60.3% in goats, 43.6% in yellow cattle, and 1% in water buffalo and horses [12]. Given that yellow cattle and water buffalo both act as important natural hosts for schistosomes in endemic areas, understanding more about the molecular mechanisms involved in worm development, as well as the pathological and immunological differences between yellow cattle and water buffalo pre and post infection with *S japonicum *will provide useful information for the design of a vaccine and its delivery procedure. However, relatively few studies on the mechanism underlying the differences in patterns of infection among hosts have been reported, and the relevant immune mechanism is not clear. In this study, yellow cattle and water buffalo were artificially infected with *S. japonicum *cercariae, and at different times post infection(0 weeks [w], 2w, 4w, 7w), the CD4^+ ^and CD8^+ ^T cell subsets and the intracellular cytokines interferon-γ(IFN-γ) and interleukin-4(IL-4) in the peripheral blood, as well as specific serum IgG antibodies, were detected and analyzed. In addition, the morphology and development rate of the worms were compared between the two host species. The study provides basic information for increasing understanding of the differences in schistosome infection between two native hosts, the susceptible host yellow cattle and the less susceptible water buffalo.

## Methods

### Animals and parasites

Six yellow cattle and six water buffalo, which were male, 15-18 months old, and free of parasitic and other infectious diseases, were used for experimental infection. The animals were purchased from areas non-endemic for schistosomes and had similar body weight. All animals were housed in covered pens, cared for by trained animal keepers and fed with hay and a commercial pelleted ration. Water and mineralized salt were available ad libitum. Cercariae of *S. japonicum *(Chinese strain) were obtained from the snail-maintenance room at Shanghai Veterinary Research Institute, Chinese Academy of Agricultural Sciences. The cercariae were shed at 20-25°C before challenge to ensure maximum vitality. The study protocol was approved by the Animal Care and Use Committee of the Shanghai Veterinary Research Insitute, Chinese Academy of Agricultural Sciences (CAAS). The use of experimental animals in this study was approved under Project A005 by the Animal Ethics Committee of the Shanghai Veterinary Research Insitute, CAAS.

### Experimental infection and the collection of blood samples

Each of the yellow cattle and water buffalo was challenged percutaneously with 1000 and 2000 cecariae of *S.japonicum*, respectively, through the upper back using the cover glass method [13]. Before infection and at 2w, 4w and 7w post infection, blood samples were collected from each animal using ordinary vacuum tubes containing sterile anticoagulant heparin. The blood samples for flow cytometry analysis were treated in 5 hours; serum separation was completed within 24 hours.

### Animal pathology observation and collection of parasite and tissue samples

The animals were sacrificed 7 weeks post infection and the parasites were perfused through the hepatic portal vein. The male and female worms were detached manually, counted, and the length of the worms was measured by the same person. The remaining worms were fixed in 4% paraformaldehyde solution after washing with phosphate buffer saline (PBS). The livers of infected hosts were harvested, dissected into 1.5 cm × 1.5 cm × 0.3 cm pieces for sectioning, and fixed in 10% neutral formalin

### Preparation of paraffin sections

The formalin-fixed samples were dehydrated in ethanol, cleared with xylene and embedded in paraffin wax. Sections (6 μm thick) were stained with HE (hematoxylin and eosin) and observed using a light microscope.

### Detection and analysis of the proportion of CD4^+ ^and CD8^+ ^T cell subsets as well as intracellular cytokines IFN-γ and IL-4 in the peripheral blood

#### Antibodies, reagents and equipment

Monoclonal antibodies specific to surface markers and intracellular cytokines, including anti-bovine CD4 FITC (mouse IgG2a:clone CC8), anti-bovine CD8 Alexa Flour 647 (mouse IgG2a:clone CC63), anti-bovine IFN-γ R.phycoerythrin (RPE) (mouse IgG1:clone CC302), anti-bovine IL4 R.phycoerythrin (RPE) (mouse IgG2a:clone CC303), anti-bovine CD4 RPE (IgG2a:clone CC8), anti-CD3 FITC(IgG1:clone CD3-12), as well as control antibodies for mouse IgG2a and mouse IgG1, were purchased from AbD Serotec (Oxford, UK). Phorbo 12-myristate 13-acetate (PMA), ionomycin, monensin, paraformaldehyde, and saponin were bought from Sigma-Aldrich (Germany); RPIM-1640 medium, FBS, erythrocyte lysis buffer, penicillin/streptomycin, and fixation and permeabilization buffer were from BD (Becton Dickinson, USA); the FACScalibur flow cytometer was obtained from BD (Becton Dickinson, USA); the CO_2 _cell incubator was from HERA GmbH (Germany).

#### Detection and analysis of the proportion of CD4^+ ^and CD8^+ ^T cell subsets

To remove red blood cells (RBCs), 0.5-1 mL heparinized whole blood was added to 15 mL erythrocyte lysis buffer, mixed for 3-5 min, centrifuged at 800-1000 rpm for 5 min, and washed twice with PBS. Vital cells were counted by means of trypan blue dye exclusion staining and adjusted to 1 × 10^9 ^cells/L. Each sample was divided into three parallel tubes with 100 μl cell suspension in each tube.

The procedure for flow cytometry (FCM) staining followed the method described by Liu [14]. Briefly, the cells of each sample were stained with anti-CD4 RPE and anti-CD3 FITC, or anti-CD8 Alexa Flour 647 and anti-CD3 FITC. The tubes were mixed thoroughly, incubated at room temperature for 20-30 min in the dark, and the percentages of the CD3 + CD4+ and CD3 + CD8+ T cell subsets were recorded using a FACScalibur flow cytometer (BD, USA). Analyses of the FCM data were performed using CellQuest software.

#### Detection and analysis of the level of intracellular cytokines IFN-γ and IL-4

Heparinized whole blood and RPMI 1640 medium were mixed in a ratio of 1:1, and RBCs were treated as mentioned above. The cell mixture was washed twice with RPMI 1640 medium and grown in complete RPMI 1640 medium containing 2 mM L-glutamine, 100 U of penicillin/mL, and 0.1 mg/mL streptomycin in an incubator at 37°C with 5% CO_2_. Intracellular staining was performed as described previously [15]. The cells were adjusted to a concentration of 1 × 10^7^/mL in complete RPMI 1640 medium. For cytokine assays, the procedures were performed according to the manufacturer's protocol. Briefly, about 2 × 10^6 ^cells were set in each tube, the cells were stimulated in the presence of PMA (25 μg/L, ionomycin (1 mg/L), and monensin (2 mg/L) for 6 h, then incubated with surface marker antibodies in the dark for 10 min. Fixation and permeabilization buffer were added, and the cells were washed in PBS again and stained with intracellular antibodies at room temperature for 20-30 min in the dark. Finally the cells were washed and detected by flow cytometry.

#### Detection and analysis of specific serum IgG antibodies

The level of specific antibodies against schistosome egg antigen (SEA) was determined by enzyme-linked immunosorbent assay(ELISA). The SEA was prepared from the livers of rabbits infected with *S. japonicum *as described before; the concentration of SEA was measured using the bicinchoninic acid (BCA) method (Pierce Europe B.V., The Netherlands). Microtiter plates (Maxisorp, Nunc, UK) were coated with SEA (10 μg/mL) in 0.1 M carbonate buffer, pH 9.6 (100 μL/well) and incubated overnight at 4°C, before blocking with 8% pig serum in PBS with 0.05% Tween 20 at 37°C for 1 h. Subsequently, 100 μL of test sera diluted at 1:200 was added in triplicate and incubated for 60 min at 37°C. Following this, 100 μL of horseradish peroxidase-conjugated rabbit anti-cow IgG (Dakocytomation, Cambridgeshire, UK) was added with different dilution and incubated for 60 min, then 100 μL of the soluble type TMB substrate solution (Tiangen, Beijing) was added and incubated for 10 min. The reaction was stopped by addition of 50 μL of 2 M sulfuric acid. The optical density (OD) was read at 450 nm in an ELISA reader (BioTek, USA). After each step, the plates were washed with PBS-0.1% Tween 20 three times for 5 min each.

### Statistical analysis

Statistical differences between cattle and water buffalo were determined using a Student's *t*-test and expressed as *p*-values. A value of *p *< 0.05 was considered statistically significant.

## Results

### Histopathological differences in the livers of yellow cattle and water buffalo infected with *S. Japonicum*

**Figure 1:**
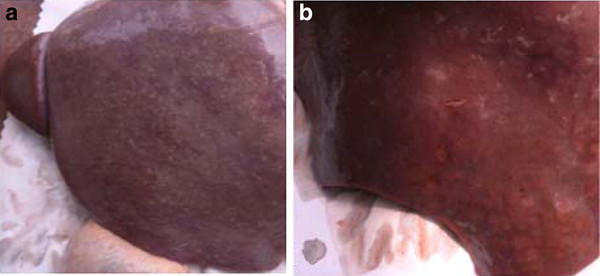
**Comparison of liver pathological changes**. Livers from two natural hosts at 7w post infection: (A) yellow cattle; (B) water buffalo.

Yellow cattle and water buffalo were sacrificed 7 weeks post infection and the differences in livers between yellow cattle and water buffalo were observed. The results revealed that the livers from yellow cattle were dark red with many white egg nodules, composed of many egg-granulomas, while the livers from water buffalo were red with few or no egg nodules (Figure 1).

**Figure 2:**
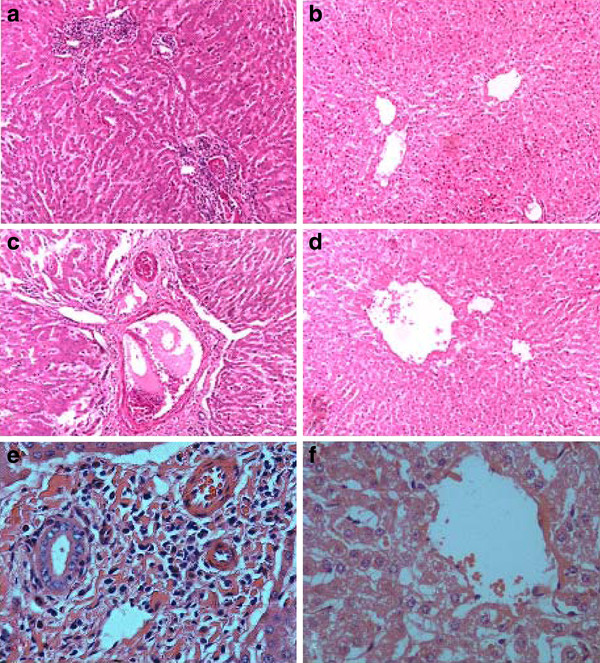
**Micrographs of HE staining of the liver tissue sections**. The liver tissue sections from two natural hosts: yellow cattle (A, C, E) and water buffalo (B, D, F), A, B, C, D are 10 × 10 magnification, E and F are 10 × 40 magnification.

The histological sections from livers of infected yellow cattle showed that some hepatocytes displayed mild swelling, a large number of inflammatory cells were seen to be infiltrating and aggregating, including eosinophils and lymphocytes, and typical striped eosinophilic deposits were seen. This is known as the Hoeppli phenomenon [16], and is formed by immuno-precipitation of egg antigens and host antibodies in the liver. Moderate to severe fibrous perihepatitis and a number of white migratory tracts in the hepatic surface and parenchyma were observed, which were composed of an abundant infiltrate of eosinophils and macrophages, some of them with a proliferation of fibrous connective tissue (Figure 2, A, C, E). Compared with the yellow cattle, the structural integrity of the hepatic lobules was intact in the livers of infected water buffalo, there was actinomorphous distribution of hepatic cord centered on central veins, polygonal hepatocytes, no edema, no inflammatory cell infiltrated, and only scattered neutrophils and monocytes (Figure 2, B, D, F).

### Differences in morphology and development rate between worms from yellow cattle and water buffalo

**Figure 3:**
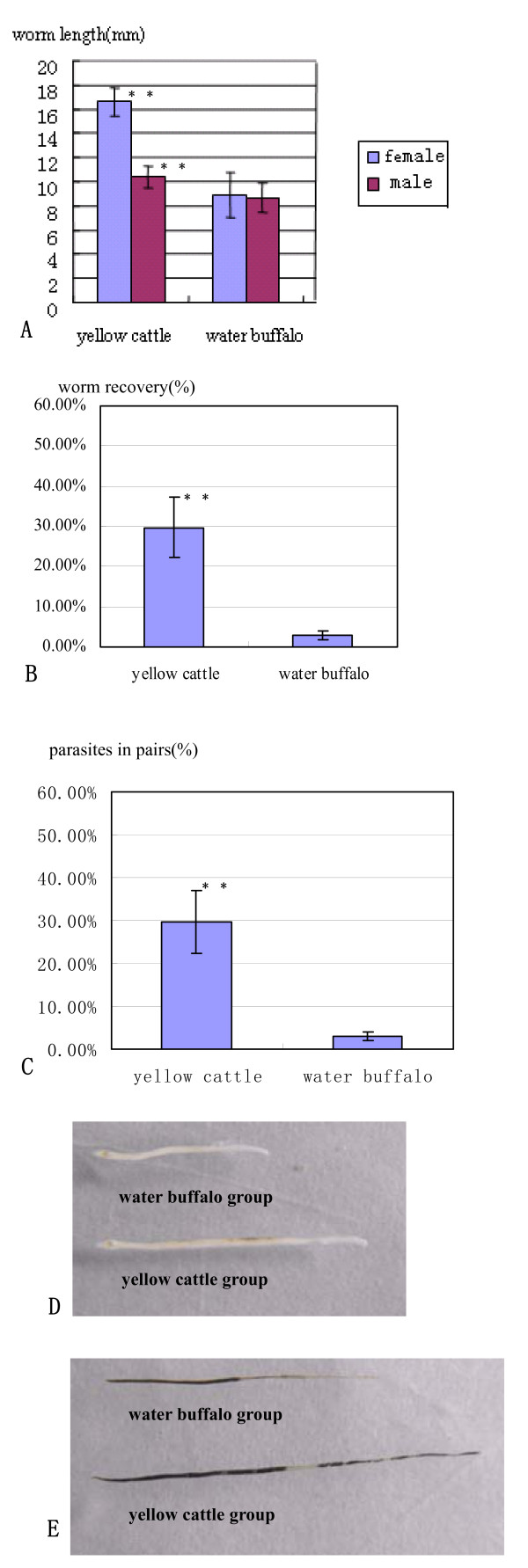
**Worms were collected from the two groups at 7w post infection**. (A) Worm length compared between the two groups; (B) the worm recovery rate in each group was calculated from the number of worms perfused at 7w post infection versus the number of cercariae infected, respectively; (C) comparison of the numbers of paired worms versus the total worms collected from yellow cattle and water buffalo; (D) male worms, the upper worm was from water buffalo and the lower worm was from yellow cattle; (E) female worms, upper from water buffalo and lower from yellow cattle. Level of significance when compared to water buffalo group, **p < 0.01. The number of worms compared per group was at least n = 50 for females and males, respectively, and included worms from all animals.

The animals were sacrificed and the parasites were perfused through the hepatic portal vein 7 weeks post infection. The average worm recovery rate from water buffalo was only 2.9%, around one-tenth of that of yellow cattle (29.7%). Attenuated parasite development was observed in water buffalo, which was evidenced by reduced numbers of paired worms and changes in morphology. The percentage of paired worms from water buffalo was significantly fewer than those from yellow cattle (*p *< 0.01). The length and width of the worms from the two hosts were compared, and this revealed that no significant difference was seen in the width, but both male and female adult worms from yellow cattle were significantly longer than those from water buffalo (*p *< 0.01), and the average length of female worms from yellow cattle was nearly twice as long as those from water buffalo (see Figure 3).

### Comparison of the proportion of CD4^+ ^and CD8^+ ^T cell subsets in yellow cattle and water buffalo infected with *S. Japonicum*

**Figure 4:**
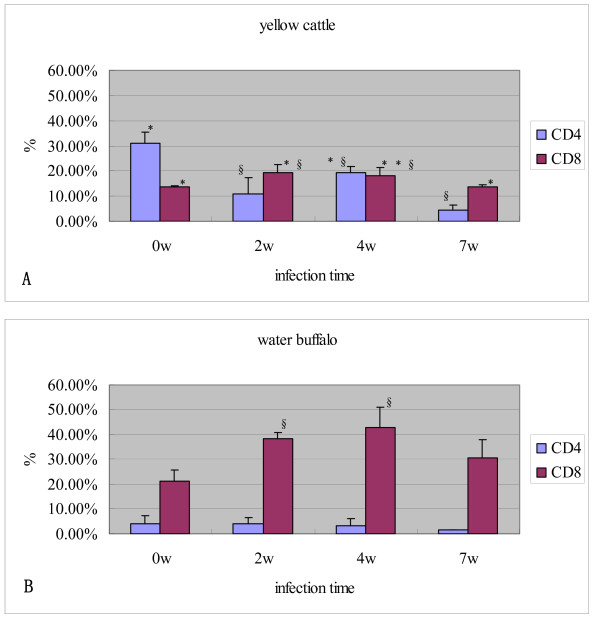
**Percentage of CD4 and CD8 T cells detected by flow cytometry pre and post infection**. (A) yellow cattle; (B) water buffalo. Level of significance when compared to water buffalo group at the same time, *p < 0.05, **p < 0.01. Level of siginificance when compared to 0w in each host, § p < 0.05.

The proportion of CD4^+ ^and CD8^+ ^T cell subsets in the peripheral blood from animals before and after challenge with *S. japonicum *was determined using flow cytometry. The results showed that the percentage of CD4^+ ^T cells in yellow cattle was always significantly higher than that in water buffalo from pre-infection to 7w post infection. The proportion of CD4^+ ^T cells was significantly decreased after yellow cattle were challenged with schistosomes, and was significantly decreased at 2w post infection, increased slightly at 4w post infection, then decreased again at 7w post infection in yellow cattle. However, the change in the proportion of CD8^+ ^T cells was not as significant as that seen in CD4^+ ^T cells before and post infection with schistosomes in yellow cattle. The results also showed that the percentage of CD8^+ ^T cells in water buffalo was significantly higher than that in yellow cattle from pre-infection to 7w post infection. The proportion of CD8^+ ^T cells was increased after water buffalo were challenged with schistosomes; it had increased significantly at 2w post infection, and continued to increase at 4w post infection, then decreased slightly at 7w post infection. A lower level of CD4^+ ^T cells and no significant change was observed before and after challenge with schistosomes in water buffalo (Figure 4).

**Figure 5:**
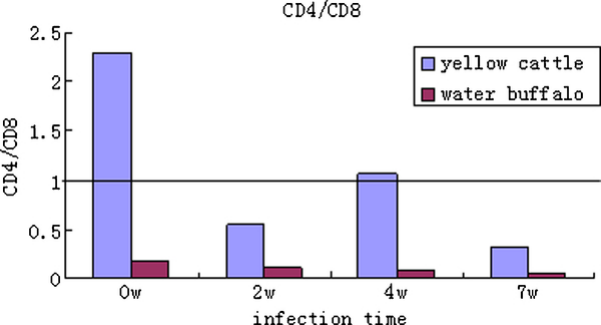
**The ratio of CD4/CD8 cells in yellow cattle and water buffalo pre and post infection**.

The ratio of CD4/CD8 cells in yellow cattle and water buffalo pre and post infection with schistosomes were compared and analyzed. The ratio was always higher in yellow cattle than in water buffalo. The ratio of CD4/CD8 cell was significantly decreased when yellow cattle were infected with *S. japonicum*, but it was lower and no significant decrease was observed in water buffalo (Figure 5).

### Comparison of the level of intracellular cytokines IFN-γ and IL-4 between yellow cattle and water buffalo infected with *S. Japonicum*

**Figure 6:**
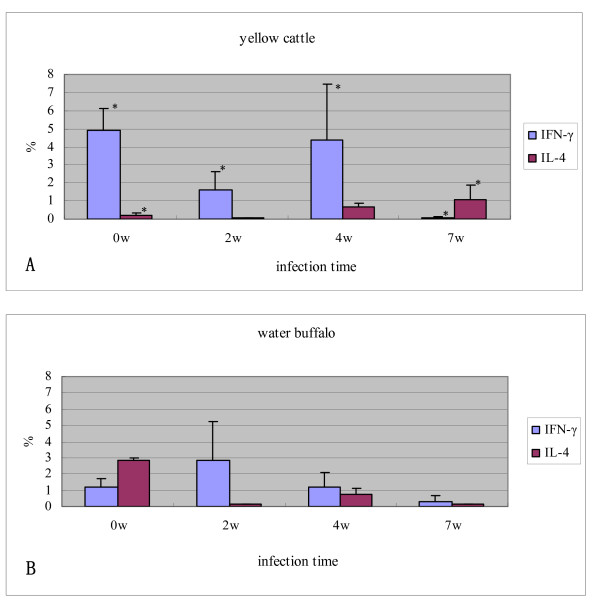
**Percentage of IFN-γ and IL-4 positive cells detected by flow cytometry pre and post infection**. yellow cattle; (B) water buffalo. Level of significance when compared to water buffalo group at the same time, *p < 0.05.

The proportion of intracellular cytokines IFN-γ and IL-4 produced by peripheral blood cells in yellow cattle and water buffalo pre and post infection with *S. japonicum *were detected. At 2w post infection, the percentage of IFN-γ decreased significantly in yellow cattle, then increased to a level near to the pre-infection level at 4w post infection, and decreased to a very low level at 7w post infection. The level of IL-4 was very low pre-infection and at 2w post infection in yellow cattle, and a low IL-4 level was detected at 4w and 7w in yellow cattle challenged with schistosomes. The level of IFN-γ in pre-infection water buffalo was much lower than that in yellow cattle; the level of the cytokine increased in water buffalo 2w post infection, then decreased to a similar level to that in pre-infected animals, and continued to decrease to an even lower level at 7w post infection. The IL-4 level was much higher in pre-infected water buffalo than in yellow cattle, but subsequently the cytokine decreased to a very low level at 2w post infection, increased at 4w and decreased at 7w post infection in water buffalo (Figure 6).

### Specific antibody response post infection

**Figure 7:**
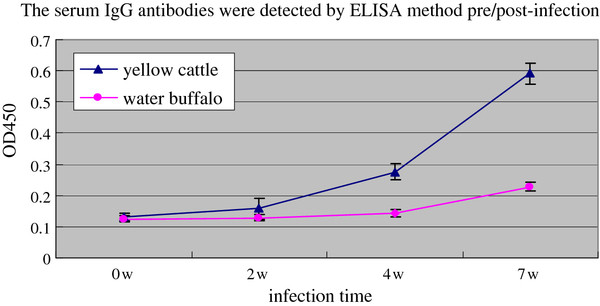
**The specific serum IgG antibody response in yellow cattle and water buffalo detected by ELISA**.

The specific serum IgG antibody was detected by ELISA in yellow cattle and water buffalo pre and post infection with *S. japonicum*. The results showed that the antibody response in water buffalo was at a low level until 7w post infection; while the level of SEA specific IgG antibodies was increased gradually in yellow cattle after infection with schistosomes in yellow cattle and and reached a higher level at 7w post infection (Figure 7).

## Discussion

Increased understanding of the development of schistosomes in yellow cattle and water buffalo, and elucidation of the related molecular mechanisms involved in *S. japonicum *infection caused by these two important natural hosts will provide useful information for the design and delivery of a vaccine against schistosomiasis. In this study, we confirmed that yellow cattle were more suited to the development and survival of schistosomes than water buffalo. Studies have shown that in some animal hosts, such as rats, pigs, and water buffalo, in the period after *S. japonicum *infection, the worm burden drops sharply, mainly owing to parasite clearance by the immune system and non-immune system factors, called the self-cure phenomenon [12,17,18]. This phenomenon is not found in mice, yellow cattle and some other schistosome-susceptible hosts. Several epidemiological surveys have reported that the natural infection of yellow cattle with schistosomiasis is obviously more severe than that of water buffalo [5,19-21]. But water buffalo reared in endemic areas have more chances and longer time to contact with the contaminated water, that is, there are more chances for water buffalo to get infection and re-infection than yellow cattle and other animals, so although water buffalo are less suitable for the development of *S. japonicum*, they still retained a higher infection rate for this parasite. For many diseases, genetically determined host susceptibility can play a role in the clinical outcome. The pathogenesis of schistosomiasis and the apparent involvement of the immune response in both susceptibility and resistance have proved complex and difficult to understand, and therefore more research on these topics is required. The characterization of schistosome responses to host factors and the host factors that elicit/prevent developmental responses in schistosomes is important and also warrants further investigation [22,23].

The tissue immune response and pathological damage caused by *S. japonicum *infection were observed in this study. The results revealed that the disease was much more serious in yellow cattle than in water buffalo; it seems that yellow cattle produce a stronger immune response than water buffalo, but the molecular mechanism for this phenomenon is still unknown.

It has been reported that schistosomes require signals from the host's immune system in order to develop fully into egg-producing adults [24,25]. In the present study, we observed that the percentage of CD4^+ ^T cells or the ratio of CD4/CD8 in yellow cattle was significantly higher than those in water buffalo from pre-infection to 7w post infection (Figure 4 and 5). This may be one explanation why schistosomes grow better in yellow cattle than in water buffalo. It has been supposed that a highly evolved relationship exists between schistosomes and their hosts that may include parasite exploitation of host endocrine and immune signals [26,27]. Recent studies have confirmed, further, that *S. mansoni *failed to receive appropriate signals from the host immune system after infection of immunodeficient mice (RAG-1^-/-^), which resulted in the appearance of attenuated forms. Hepatic CD4^+ ^T lymphocyte populations were identified as an integral component of the immune signal recognized by the parasite. Reconstitution with CD4^+ ^cells rescued schistosome development during prepatency, which resulted in larger parasites, higher levels of pairing, and dramatically increased levels of egg production. Depletion of CD4^+ ^cells but not CD8^+ ^cells from wild-type mice in vivo by the administration of specific antibodies reduced egg production by the parasites. Therefore, the authors suggested that CD4^+ ^T lymphocytes were responsible for promoting early schistosome development [24]. CD4^+ ^T lymphocytes have also been found to be necessary for the development of a parasitic protozoan: Houpt et al. reported that the depletion of CD4^+ ^cells diminished both parasite burden and inflammation significantly in the mouse model of amebic colitis [28]. Lamb et al. further found that schistosomes co-opt innate immune signals to facilitate worm development, and that CD4^+ ^T cells influence the parasites indirectly by modulating monocyte/macrophage function [29]. In this study, our results support the hypothesis proposed by Davies et al. and other investigators, and suggest that CD4^+ ^T lymphocytes might be an integral component of the immune response and might related with worm development in yellow cattle, a natural host of the schistosome, but more investigations should be carried out to confirm it and whether susceptibility is correlated with the differences in the innate immune system between yellow cattle and water buffalo also needs further investigation.

Our observations also showed that the proportion of CD4^+ ^T cells in yellow cattle decreased, the CD8^+ ^T cells proportion in water buffalo increased, and the CD4/CD8 ratios were decreased in both species after challenge with schistosomes. The observations in yellow cattle were consistent with a previous report on mice, another schistosome-sensitive host [30]. Subsets of CD4^+ ^T cells are characterized by their cytokine-production profiles. Th1 cells produce primarily IFN-γ, and generally provide protection against intracellular pathogens, whereas Th2 cells produce mainly IL-4, IL-5 and IL-13, and are important for immunity against helminth parasites. During the schistosome infection process, the immune response includes at least three stages. In the first 3-5 weeks, mainly immature parasites migrate inside the host, which shows a dominant Th1-type response. In the following 5-6 weeks, the worms mature and begin spawning in pairs, and the immune response changes significantly: the Th1-type response decreases, but the Th2-type response becomes significantly enhanced. This response is stimulated mainly by the egg antigen. In chronic infection, after a long period of time with the continuous generation of parasite eggs, the Th2-type response is altered by the eggs and the egg granulomas become smaller than before [31]. A study in mice found that the Th2-type cell-mediated granulomatous response appears to protect liver cells, but severe fibrosis occurs during the development of human schistosomiasis [32]. From the acute phase to the chronic phase, the immune response against the infection changes from the Th1-type to the Th2-type, and induces a significant increase of CD4^+ ^CD25^+ ^regulatory T cells, which have a broad immune suppression function [33]. In this study, we observed that, in yellow cattle, the IFN-γ level was higher pre-infection and at 4 weeks post infection, then decreased significantly and was at a very low level 7 weeks post infection. In addition, the level of IL-4 was very low pre-infection and 2w post infection; IL-4 was also expressed at a lower level at 4w but had increased at 7w post challenge with schistosomes. Th1 polarized immunity during the early phase of infection shifted to Th2 polarized immunity at 5-6 weeks. This was shown clearly in schistosome-infected yellow cattle, and was similar to the shift observed in other schistosome-susceptible hosts such as humans and mice [31]. Previous reports have suggested that CD8^+ ^T cells may erode the Th2 cell population steadily during chronic infection, which results in a reduced inflammatory reaction in murine schistosomiasis [34]. In water buffalo, the percentage of CD8^+ ^T cells was always significantly higher than that in yellow cattle; the level of IFN-γ was low in pre-infected animals, increased during early infection (2w), then decreased at 4w and 7w post infection. The IL-4 level was high in pre-infected water buffalo, then decreased post infection, and was at a very low level 7w post infection. No obvious shift of Th1-type to Th2-type polarized immunity was observed in water buffalo after infection with schistosomes. In addition, there were few egg granulomas and little pathological damage was seen in schistosome-infected water buffalo.

## Conclusion

In this study, the development of schistosomes in yellow cattle and water buffalo, as well as the pathological and immunological differences between the two hosts post infection with *S. japonicum *were compared and have been discussed. We confirmed that yellow cattle were more suited to the development of *S. japonicum *than water buffalo, and more serious pathological damage was observed in yellow cattle than in water buffalo after animals were infected with schistosomes. Immunological analysis suggested that CD4^+ ^T cells might be an integral component of the immune response and might associate with worm development in yellow cattle and Th1 dominated the immune response during early infection, then the response shifted to Th2-type polarized immunity at 6-7 weeks, but no obvious shift of Th1-type to Th2-type polarized immunity was observed in schistosome-infected water buffalo. These results provide valuable information for increasing understanding of why schistosomes grow better and cause more serious pathological damage in yellow cattle than in water buffalo, and for the design of vaccines for the control of this important zonnnotic disease. Further elaboration of the mechanism of self-cure phenomen in water buffalo could be important for schistosome vaccine research and application.

## Competing interests

The authors declare that they have no competing interests.

## Authors' contributions

Conceived and designed the experiment: YJM and LJJ. Performed the experiments: YJM, FZQ, SYJ, LJM, HY, LH, LK. Contributed materials/revised manuscript: FXG, FZQ, YCX. Wrote the paper: YJM and LJJ. All authors read and approved the final manuscript.
